# Supplementary material for: Femora nutrient foramina and aerobic capacity in giant extinct xenarthrans
Source: PeerJ. 2024 Aug 7;12:e17815. doi: 10.7717/peerj.17815 (PMC11316464; doi:10.7717/peerj.17815)
Supplement: Supplemental Information 1 — Supplementary Material including expanded results, Fig. S1, Table S1, and references. [file peerj-12-17815-s001.pdf]

Electronic Supplementary Material for:

Femora Nutrient Foramina and Aerobic Capacity in Giant Extinct Xenarthrans

Luciano Varela[1,2]*, P. Sebastián Tambusso[1,2], Richard A. Fariña[1,2]

[1] Departamento de Paleontología, Facultad de Ciencias, Universidad de la República, Iguá 4225, 11400 Montevideo, Uruguay.

[2] Servicio Académico Universitario y Centro de Estudio Paleontológicos (SAUCE-P), Universidad de la República, Departamento de Canelones, Santa Isabel s/n, 91500, Sauce, Uruguay.

* Corresponding author: Luciano Varela, E-mail: luciano.lvr@gmail.com. ORCID: 0000-0002-9481-6558

Contents

Expanded Results. Results for the analyses using the morphological tree and Q_i.

Figure S1. Correlation between Q_i and $\dot{Q}$.

Table S1. Data used in the study.

References

Expanded Results

Using Q_i instead of $\dot{Q}$

The estimated values for Q_i showed a highly significant correlation with $\dot{Q}$, with a $R^2 = 0.97$ and $p < 0.001$ (Fig. S1). Furthermore, the different analyses performed using Q_i instead of $\dot{Q}$ showed similar results. In particular, the interaction coefficient was not significant (p = 0.082) and the intercept was significantly different between the Epitheria and Xenarthra (p < 0.001), while the giant fossil xenarthra showed no significant differences with the Epitheria (p = 1), but a significant difference with the xenarthra (p < 0.001). The Epitheria and the fossil giant xenarthrans showed higher flow values for the same body mass, having flows ~2 times higher than the extant xenarthrans. Further analyses estimating MMR values provided similar results, supporting the same conclusions as the previous analyses, but specific values are not discussed since they are considered less reliable.

Using a morphological phylogenetic tree

On the other hand, the analyses considering a topology based only on morphological data provided results compatible with those using the phylogenetic tree based on the total evidence analysis. In particular, the interaction coefficient was not significant (p = 0.187) and the intercept was significantly different between the Epitheria and Xenarthra (p < 0.001), while the giant fossil xenarthra showed no significant differences with the Epitheria (p = 1), but a significant difference with the xenarthra (p = 0.003). The Epitheria and the fossil giant xenarthrans showed higher flow values for the same body mass, having flows ~2 times higher than the extant xenarthrans. Further analyses estimating MMR values provided similar results, supporting the same conclusions as the previous analyses, but specific values are not discussed since they are considered less reliable.

Figure S1.

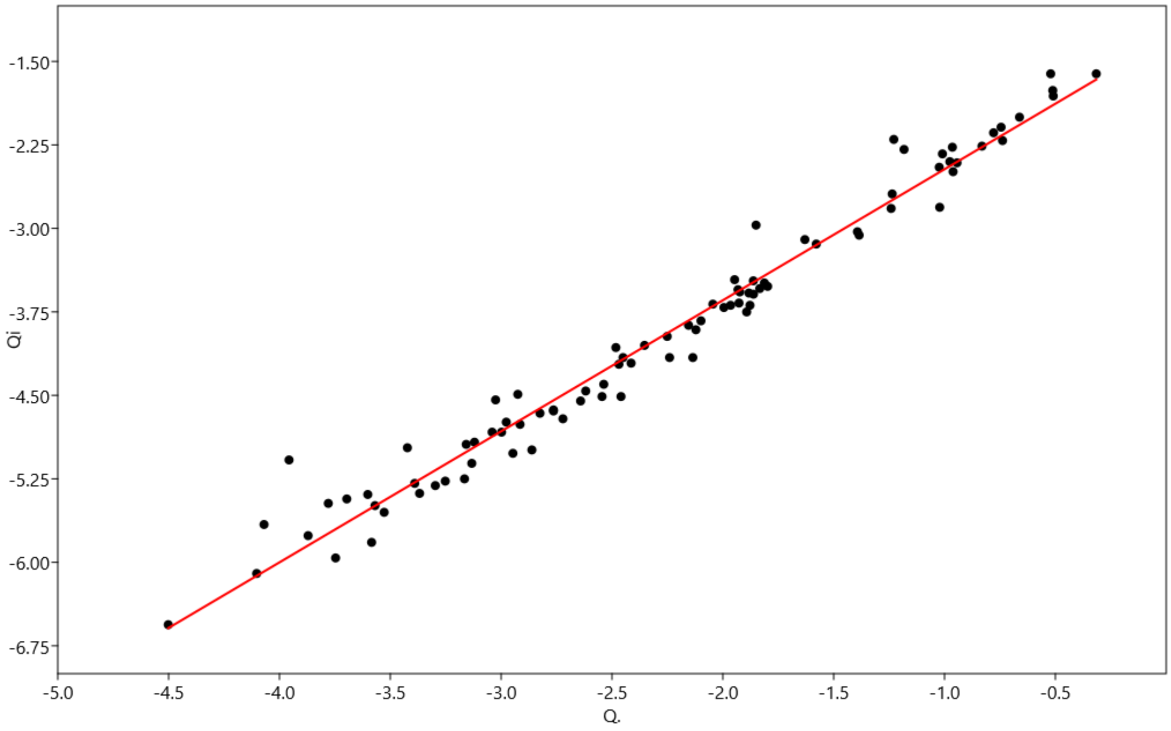

Taxa	Cat. Number	Body Mass (g)	MMR (ml O2 h-1)	Femur Length (mm)	Foramen Diameter (mm)	Foramen 2 Diameter (mm)	Blood flow rate (Q; cm3 s-1)	Blood flow rate Index (Qi = r4/L)	Source
Bradypus torquatus	MHND w/n	3900		98.5	0.204		1.79E-04	1.10E-06	Marquet and Cofre, 1999
Bradypus tridactilus	MHND 145	3850	1412.5	101.8	0.306		5.60E-04	5.38E-06	Irving et al., 1942 (MMR); Silva and Downing, 1995
Cabassous unicinctus	MHND 84	3200		70.5	0.282		4.46E-04	5.61E-06	Emmons, 1990
Cabassous unicinctus	MHND 83	3200		79.3	0.448		1.59E-03	3.17E-05	Emmons, 1990
Cabassous unicinctus	MHND 13890	3200		78.3	0.386		1.06E-03	1.77E-05	Emmons, 1990
Catonyx cuvieri	MHND 228	598000		448	2.739		1.62E-01	7.85E-03	Dantas 2022
Catonyx cuvieri	MHND 2565	598000		385	1.988		7.44E-02	2.54E-03	Dantas 2022
Catonyx cuvieri	MHND 2566	598000		406	1.757		5.49E-02	1.47E-03	Dantas 2022
Catonyx cuvieri	MHND 2563	598000		396	2.429		1.21E-01	5.49E-03	Dantas 2022
Chaetophractus vallerosus	MHND 215	1030		66	0.336		7.25E-04	1.21E-05	Silva and Downing, 1995
Chaetophractus vallerosus	MHND 68	1030		64.5	0.326		6.67E-04	1.09E-05	Silva and Downing, 1995
Choloepus sp.	MHN-TL 3274	6250		105.1	0.338		7.37E-04	7.76E-06	De Magalhaes and Costa, 2009
Cyclopes didactylus	MHND 114	240		36	0.112		3.15E-05	2.73E-07	White and Seymour, 2003
Dasypus hybridus	MHND 9663	1600		67.3	0.273		4.07E-04	5.16E-06	Silva and Downing, 1995
Dasypus novencintus	MHND 24	4500	8838	90	0.278		4.28E-04	4.15E-06	Boily, 2002
Dasypus punctatus	MHND 9414	19309		160	0.472	0.305	2.93E-03	1.94E-05	This study. Based on femur length
Dasypus punctatus	MHND 2162	42028		200	0.939		1.13E-02	2.43E-04	This study. Based on femur length
Euphractus sexcintus	MHND 62	4800		82.3	0.404		1.20E-03	2.02E-05	Silva and Downing, 1995
Euphractus sexcintus	MHND 12222	4800		70.3	0.367		9.24E-04	1.61E-05	Silva and Downing, 1995
Glossotherium robustum	MNHN-M w/n	1713000		510	2.772		1.67E-01	7.24E-03	Bargo et al., 2000
Glossotherium robustum	MNHN-M w/n	1713000		500	2.77		1.66E-01	7.36E-03	Bargo et al., 2000
Glyptodon reticulatus	MNHN-M w/n	862300		550	3.387		2.68E-01	1.50E-02	Fariña et al., 1998
Glyptodon reticulatus	MNHN-M 27	862300		550	3.065		2.12E-01	1.00E-02	Fariña et al., 1998
Glyptodon reticulatus	MNHN-M 1525	862300		506	2.107		8.58E-02	2.43E-03	Fariña et al., 1998
Glyptodon reticulatus	MHND w/n	862300		515	3.438		2.78E-01	1.70E-02	Fariña et al., 1998
Glyptodon reticulatus	MNHN-F PAM 158	862300		513	3.117		2.20E-01	1.15E-02	Fariña et al., 1998
Glyptodon reticulatus	MNHN-F PAM 162	862300		555	3.4		2.71E-01	1.50E-02	Fariña et al., 1998
Glyptodon reticulatus	MPAC 1868	862300		468	2.197		9.50E-02	3.11E-03	Fariña et al., 1998
Glyptodon reticulatus	MNHN-M 27	862300		500	3.204		2.35E-01	1.32E-02	Fariña et al., 1998
Glyptodon reticulatus	MNHN-M 1003	862300		530	2.331		1.10E-01	3.48E-03	Fariña et al., 1998
Holmesina majus	MHND 472	177900		347	0.972		1.23E-02	1.61E-04	Vizcaíno et al., 2006 (Similar to H. occidentalis)
Holmesina majus	MHND 473	177900		316	1.001		1.33E-02	1.99E-04	Vizcaíno et al., 2006 (Similar to H. occidentalis)
Lestodon armatus	MPAC w/n	3397000		696	3.97		3.89E-01	2.23E-02	Fariña et al., 1998
Lestodon armatus	MNHN-M 2325	3397000		680	3.691		3.28E-01	1.71E-02	Fariña et al., 1998
Lestodon armatus	MNHN-M 155	3397000		710	3.412		2.73E-01	1.19E-02	Fariña et al., 1998
Lestodon armatus	MNHN-M w/n	3397000		720	3.023		2.05E-01	7.25E-03	Fariña et al., 1998
Lestodon armatus	MNHN-M 2790	3397000		720	4.191		4.41E-01	2.68E-02	Fariña et al., 1998
Lestodon armatus	AdV 977	3397000		690	3.443		2.79E-01	1.27E-02	Fariña et al., 1998
Macrauchenia patachonica	MNHN-F PAM 78	988000		564	2.327		1.09E-01	3.25E-03	Fariña et al., 1998
Manis javanica	MHND 13	5150		88	0.587		3.29E-03	8.43E-05	Ernest, 2003
Megatherium americanum	MHND 121	3564000		720	3.893		3.72E-01	1.99E-02	Fariña et al., 1998
Megatherium americanum	MHND 23	3564000		680	2.894		1.85E-01	6.45E-03	Fariña et al., 1998
Megatherium americanum	MNHN-F 1881.35	3564000		652	3.706		3.31E-01	1.81E-02	Fariña et al., 1998
Megatherium americanum	MPAC 898	3564000		696	3.847		3.61E-01	1.97E-02	Fariña et al., 1998
Megatherium americanum	MNHN-M 375	3564000		650	4.003		3.97E-01	2.47E-02	Fariña et al., 1998
Megatherium americanum	MNHN-M w/n	3564000		690	3.974		3.90E-01	2.26E-02	Fariña et al., 1998
Megatherium americanum	MHND 213	3564000		587	3.027		2.05E-01	8.94E-03	Fariña et al., 1998
Mylodon darwinii	AdV B-2(9)	1986000		695	2.881		1.83E-01	6.20E-03	Fariña et al., 1998
Myrmecophaga tridactyla	MHND 105	39000		211	0.9		1.01E-02	1.94E-04	Wetzel, 1985; Reis et al., 2006
Neoglyptatelus uruguayensis	MNHN-M 1642	8993		128.5	0.594		3.40E-03	6.06E-05	This study. Based on femur length
Neosclerocalyptus ornatus	MNHN-F R.191 D	300000		284	2.197		9.50E-02	5.13E-03	Fariña, 1995
Neosclerocalyptus paskoensis	MHND 170	300000		348	1.718	0.785	6.57E-02	1.56E-03	Fariña, 1995 (Similar to *N. ornatus*)
Nothrotherium maquinense	MHND 1/1845 6697	171000		255	1.04		1.47E-02	2.87E-04	Dantas, 2022
Panochthus tuberculatus	MPAC w/n	1061000		460	2.106		8.57E-02	2.67E-03	Fariña et al., 1998
Panochthus tuberculatus	MNHN-M w/n	1061000		550	2.661		1.51E-01	5.70E-03	Fariña et al., 1998
Phascolarctos cinereus	MHND 29	10250		149	0.384		1.05E-03	9.12E-06	Strahan, 1995
Priodontes maximus	MHND 100	45000		164	0.861		9.01E-03	2.09E-04	Silva and Downing, 1995
Propraopus sulcatus	MHND 11686	47000		193	0.821		7.96E-03	1.47E-04	Fariña and Vizcaíno, 1997
Scelidotherium leptocephalum	MHND 224	1057000		463	1.828		6.06E-02	1.51E-03	Bargo et al., 2000
Scelidotherium leptocephalum	MHND 63	1057000		435	2.37		1.14E-01	4.53E-03	Bargo et al., 2000
Scelidotherium leptocephalum	MHND 67	1057000		510	1.976		7.34E-02	1.87E-03	Bargo et al., 2000
Scelidotherium leptocephalum	MNHN-M w/n	1057000		465	1.227		2.24E-02	3.05E-04	Bargo et al., 2000
Tamandua tetradactyla	MHND 110	5500		98	0.293		4.96E-04	4.70E-06	Silva and Downing, 1995
Tamandua tetradactyla	MHND 111	5500		100	0.556		2.85E-03	5.97E-05	Silva and Downing, 1995
Toxodon platensis	MPAC 1846	1642000		583	3.102		2.18E-01	9.93E-03	Fariña et al., 1998
Valgipes bucklandi	MHND 3351	724000		420	1.777		5.65E-02	1.48E-03	McDonald, 2023
Valgipes bucklandi	MHND 7717	724000		420	3.438	1.952	3.85E-01	2.08E-02	McDonald, 2023
Vombatus ursinus	MHND 32	26000		128.4	0.631		3.99E-03	7.72E-05	Strahan, 1995

Taxa	Body Mass (g)	BMR (ml O2 h-1)	MMR (ml O2 h-1)	Femur Length (mm)	Foramen Diameter (mm)	Blood flow rate (Q̇; cm3 s-1)	Blood flow rate Index (Qi = r4/L)	Source
Aepyprymnus rufescens	2820	1072		103.9613	0.598972	3.47E-03	3.07E-05	Seymour et al. 2012
Alces alces	325000	51632.46		429.5	1.788895	5.74E-02	1.50E-03	Seymour et al. 2012
Antechinus minimus				26.67	0.157455	8.52E-05	2.16E-06	Seymour et al. 2012
Arctocephalus fosteri	55000			73.25	0.995107	1.31E-02	2.64E-04	Seymour et al. 2012
Arctocephalus pusillus	75000			128.4125	1.024448	1.41E-02	1.08E-03	Seymour et al. 2012
Bettongia lesueur	1300			78.0125	0.437793	1.50E-03	2.19E-05	Seymour et al. 2012
Bettongia penicillata	1018	561	11682	79.61	0.405743	1.22E-03	1.73E-05	Seymour et al. 2012
Bos taurus	225000	46240	1453500	390	1.553511	4.05E-02	9.35E-04	Seymour et al. 2012
Camelus dromedarius	402000	40497	1206000	456.25	1.564337	4.12E-02	8.64E-04	Seymour et al. 2012; Sharp 2012
Canis familiaris	30625		258365	192	1.25	2.34E-02	7.95E-04	Koteja 1987; Seymour et al. 2012; Genoud et al. 2018
Capra hircus	45000	12660	80682	196	0.959452	1.19E-02	2.70E-04	Seymour et al. 2012
Cervus eldii				276.5	1.073694	1.59E-02	3.03E-04	Seymour et al. 2012
Dama dama	70000			249.5	1.059788	1.54E-02	3.27E-04	Seymour et al. 2012
Dasyuroides byrnei	92	72	1435.2	33.6925	0.184581	1.35E-04	1.76E-06	Seymour et al. 2012
Dendrolagus bennettianus	9300			154.5	0.559949	2.90E-03	4.00E-05	Seymour et al. 2012
Dorcopsis luctuosa	3570			130.25	0.328771	6.83E-04	5.65E-06	Seymour et al. 2012
Elephas maximus	4545400	409500	3818136	1010	4.358905	4.83E-01	2.47E-02	Seymour et al. 2012; Langman et al. 2012
Equus asinus	177500	29288		356.5	2.320284	1.09E-01	5.43E-03	Seymour et al. 2012
Equus caballus	675000	65000	3360300	463.75	3.55839	3.01E-01	2.45E-02	Seymour et al. 2012
Felis catus	4600	2048		109.1	0.37821	1.00E-03	1.48E-05	Seymour et al. 2012
Gazella dorcas	21370			154	0.718082	5.61E-03	1.08E-04	Seymour et al. 2012
Giraffa camelopardalis	750000			435	2.294895	1.06E-01	3.99E-03	Seymour et al. 2012
Hydrurga leptonyx	380000			139.25	0.939644	1.13E-02	3.50E-04	Seymour et al. 2012
Hypsiprymnodon moschatus	500			62.4	0.235702	2.70E-04	3.25E-06	Seymour et al. 2012
Isoodon obesulus	717	222	5731.8	62.035	0.369789	9.44E-04	2.91E-05	Seymour et al. 2012
Lama glama	100000	26450		290	1.309923	2.64E-02	7.23E-04	Seymour et al. 2012
Lasiorhinus latifrons	29917	2992		132.55	0.52217	2.41E-03	3.46E-05	Seymour et al. 2012
Leporillus conditor	315			41.5025	0.394898	1.13E-03	9.64E-06	Seymour et al. 2012
Lepus capensis	3030			119.5	0.461101	1.72E-03	2.37E-05	Seymour et al. 2012
Lepus europaeus	4175			124.55	0.461117	1.72E-03	2.27E-05	Seymour et al. 2012
Lobodon carcinophagus	215000			111.72	1.809045	5.90E-02	6.28E-03	Seymour et al. 2012
Macroderma gigas	148	139		43.47	0.15335	7.89E-05	8.03E-07	Seymour et al. 2012
Macropus agilis	12000			184.5	0.511888	2.28E-03	2.81E-05	Seymour et al. 2012
Macropus greyi	10000			145.975	0.656686	4.43E-03	8.84E-05	Seymour et al. 2012
Macropus irma	8000			154.5	0.6231	3.86E-03	6.10E-05	Seymour et al. 2012
Macropus parryi	12000			195.5	0.556234	2.85E-03	3.06E-05	Seymour et al. 2012
Macropus robustus	29300	8100		193.5	0.95238	1.17E-02	2.81E-04	Seymour et al. 2012
Macropus rufus	32490	5861	304380	250.75	0.804733	7.56E-03	1.23E-04	Seymour et al. 2012; Dawson et al. 2004
Mucropus fuliginosus	30000			267.125	1.013924	1.37E-02	2.55E-04	Seymour et al. 2012
Ornithorhynchus anatinus	693	194		43.22	0.229529	2.50E-04	4.09E-06	Seymour et al. 2012
Oryctolagus cuniculus	1590	715	6750	84.29167	0.341464	7.58E-04	1.20E-05	Seymour et al. 2012
Ovis aries	21150	10200	60822	196.5	1.013363	1.37E-02	3.37E-04	Seymour et al. 2012
Panthera pardus	41400			244	0.956388	1.18E-02	2.16E-04	Seymour et al. 2012
Perameles gunnii	837	420		41.8	0.294769	5.04E-04	4.90E-06	Seymour et al. 2012
Petaurus breviceps	127	90		40.75625	0.212531	2.01E-04	3.70E-06	Seymour et al. 2012
Phascolarctos cinereus	6528.74	1034		155	0.59702	3.44E-03	4.16E-05	Seymour et al. 2012
Potorous tridactylus	976	416	7344	79.0275	0.243785	2.96E-04	2.82E-06	Seymour et al. 2012
Pseudocheirus peregrinus	916	431		59.0375	0.232818	2.60E-04	1.53E-06	Seymour et al. 2012
Rattus lutreolus	109	63		28.955	0.198646	1.66E-04	3.36E-06	Seymour et al. 2012
Ratus fuscipes	76	84		27.1075	0.172308	1.10E-04	8.24E-06	Seymour et al. 2012
Sus scrofa	55300	8250	103896	225.5	0.924633	1.08E-02	2.05E-04	Seymour et al. 2012
Tachyglossus aculeatus	2725	431	3955.8	59.295	0.265737	3.78E-04	1.08E-05	Seymour et al. 2012
Tapirus indicus	250000			327.5	2.222953	9.78E-02	4.68E-03	Seymour et al. 2012
Tetracerus quadricornis	19000			178	0.781581	7.00E-03	1.34E-04	Seymour et al. 2012
Thylacinus cynocephalus	29999.99			206.5	0.424493	1.38E-03	1.02E-05	Seymour et al. 2012
Ursus arctos	233000	20790.6		356.5	1.185236	2.05E-02	3.46E-04	Seymour et al. 2012
Ursus arctos				268.5	0.844623	8.57E-03	1.19E-04	Seymour et al. 2012
Vombatus ursinus	22500			148.4125	1.00501	1.34E-02	6.09E-05	Seymour et al. 2012
Vulpes vulpes	4440	2442	50364	127.775	0.603721	3.55E-03	6.88E-05	Seymour et al. 2012

References

Bargo, M. S., Vizcaíno, S. F., Archuby, F. M., & Blanco, R. E. (2000). Limb bone proportions, strength and digging in some Lujanian (Late Pleistocene-Early Holocene) mylodontid ground sloths (Mammalia, Xenarthra). Journal of Vertebrate Paleontology, 20(3), 601-610.

Boily, P. (2002). Individual variation in metabolic traits of wild nine-banded armadillos (Dasypus novemcinctus), and the aerobic capacity model for the evolution of endothermy. Journal of Experimental Biology, 205(20), 3207-3214.

Dantas, M. A. (2022). Estimating the body mass of the late Pleistocene megafauna from the South America Intertropical Region and a new regression to estimate the body mass of extinct xenarthrans. Journal of South American Earth Sciences, 119, 103900.

Dawson, T. J., Mifsud, B., Raad, M. C., & Webster, K. N. (2004). Aerobic characteristics of red kangaroo skeletal muscles: is a high aerobic capacity matched by muscle mitochondrial and capillary morphology as in placental mammals?. Journal of Experimental Biology, 207(16), 2811-2821.

De Magalhaes, J. P., & Costa, A. J. (2009). A database of vertebrate longevity records and their relation to other life-history traits. Journal of evolutionary biology, 22(8), 1770-1774.

Emmons, L. H., & Feer, F. (1990). Neotropical rainforest mammals: a field guide. Chicago: University of Chicago Press .

Ernest, S. M. (2003). Life history characteristics of placental nonvolant mammals: ecological archives E084-093. Ecology, 84(12), 3402-3402.

Fariña, R. A. (1995). Limb bone strength and habits in large glyptodonts. Lethaia, 28(3), 189-196.

Fariña, R. A., & Vizcaino, S. F. (1997). Allometry of the bones of living and extinct armadillos (Xenarthra, Dasypoda). Zeitschrift fur Saugetierkunde, 62, 65-70.

Fariña, R. A., Vizcaíno, S. F., & Bargo, M. S. (1998). Body mass estimations in Lujanian (late Pleistocene-early Holocene of South America) mammal megafauna. Mastozoología Neotropical, 5(2), 87-108.

Genoud, M., Isler, K., & Martin, R. D. (2018). Comparative analyses of basal rate of metabolism in mammals: data selection does matter. Biological Reviews, 93(1), 404-438.

Irving, L., Scholander, P. F., & Grinnell, S. W. (1942). Experimental studies of the respiration of sloths. Journal of Cellular and Comparative Physiology, 20(2), 189-210.

Koteja, P. (1987). On the relation between basal and maximum metabolic rate in mammals. Comparative Biochemistry and Physiology. A, Comparative physiology, 87(1), 205-208.

Langman, V. A., Rowe, M. F., Roberts, T. J., Langman, N. V., & Taylor, C. R. (2012). Minimum cost of transport in Asian elephants: do we really need a bigger elephant?. Journal of Experimental Biology, 215(9), 1509-1514.

Marquet, P. A., and H. Cofre. 1999. Large temporal and spatial scales in the structure of mammalian assemblages in South America: a macroecological approach. Oikos 85:299–309.

McDonald, H. G. (2023). A tale of two continents (and a few islands): Ecology and distribution of Late Pleistocene sloths. Land, 12(6), 1192.

Reis, N., Peracchi, A., Pedro, W. et al. (2006). Mamíferos do Brasil. 1. ed. Curitiba: Governo do Paraná, SEMA, SBZ.

Seymour, R. S., Smith, S. L., White, C. R., Henderson, D. M., & Schwarz-Wings, D. (2012). Blood flow to long bones indicates activity metabolism in mammals, reptiles and dinosaurs. Proceedings of the Royal Society B: Biological Sciences, 279(1728), 451-456.

Sharp, N. C. (2012). Animal athletes: a performance review. Veterinary Record, 171(4), 87-94.

Silva, M., & Downing, J. A. (1995). The allometric scaling of density and body mass: a nonlinear relationship for terrestrial mammals. The American Naturalist, 145(5), 704-727.

Strahan, R., (ed.) 1995. The Mammals of Australia. Revised Edition. Reed New Holland, Sydney. 756 pp.

Vizcaíno, S. F., Bargo, M. S., & Cassini, G. H. (2006). Dental occlusal surface area in relation to body mass, food habits and other biological features in fossil xenarthrans. Ameghiniana, 43(1), 11-26.

Wetzel, R.M. (1985). The identification and distribution of recent Xenarthra (Edentata). In: Montgomery, G.G. (Ed). The Evolution and Ecology of Armadillos, Sloths, and Vermilinguas. Washington, DC: Smithsonian Institution Press, 1985. p.5-21.

White, C. R., & Seymour, R. S. (2003). Mammalian basal metabolic rate is proportional to body mass2/3. Proceedings of the National Academy of Sciences, 100(7), 4046-4049.
